# Advances in Treatment of ATTRv Amyloidosis: State of the Art and Future Prospects

**DOI:** 10.3390/brainsci10120952

**Published:** 2020-12-09

**Authors:** Massimo Russo, Luca Gentile, Antonio Toscano, M’Hammed Aguennouz, Giuseppe Vita, Anna Mazzeo

**Affiliations:** 1 Unit of Neurology and Neuromuscular Diseases, Department of Clinical and Experimental Medicine, University of Messina, 98125 Messina, Italy; lucagentile84@yahoo.it (L.G.); atoscano@unime.it (A.T.); aguenoz.mhommed@unime.it (M.A.); vitag@unime.it (G.V.); annamazzeo@yahoo.it (A.M.); 2 Department of Medicine, Mohammed VI University of Health Sciences, UM6SS Casablanca, Morocco

**Keywords:** ATTRv, amyloidosis, hereditary polyneuropathy, TTR stabilizer, TTR silencers, gene therapy

## Abstract

Hereditary amyloid transthyretin (ATTRv) amyloidosis with polyneuropathy is a progressive disease that is transmitted as an autosomal dominant trait and characterized by multiple organ failure, including axonal sensory-motor neuropathy, cardiac involvement, and autonomic dysfunction. Liver transplantation (LT) and combined heart–liver transplantation, introduced in the 1990s, have been the only therapies for almost two decades. In 2011, tafamidis meglumine became the first specific drug approved by regulatory agencies, since then the attention toward this disease has progressively increased and several drugs with different mechanisms of action are now available. This review describes the drugs already on the market, those that have shown interesting results although not yet approved, and those currently being tested.

## 1. Introduction

Hereditary amyloid transthyretin (ATTRv) amyloidosis is a multisystemic disease of adult onset, affecting the sensorimotor nerves, heart, and autonomic function along with other organs such as gastrointestinal tract, eyes, and kidney [1]. The disease is transmitted with an autosomal dominant pattern, once thought to be restricted to an area of northern Portugal and a few other endemic areas, now documented to be a worldwide disease, with a global prevalence estimated up to 38,000 persons [2,3,4]. Early onset, defined as ≤50 years of age at symptom onset, is common in Portugal and Japan, while late-onset cases are predominant in other countries [5,6,7,8].

ATTRv amyloidosis is due to missense mutations that make the transthyretin (TTR) tetramer unstable, and then, a misfolded variant of TTR protein aggregates, generating amyloid fibrils, which are found as protein deposits in tissues [9,10]. Generally, different missense mutations may present with different phenotype (e.g., mainly neuropathic or cardiac), and sometimes even the same mutation may cause different phenotypes [6,11,12]. In peripheral nerves, amyloid fibrils cause Schwann cell damage, resulting in the predominant loss of small-fiber axons characteristic of early-onset cases, while vasculopathy may also determine the pathogenesis of the neuropathy in late-onset cases [13,14].

Once polyneuropathy (ATTR-PN) has started, its progression is rapid and evolves into three progressive stages [10,15]:

- In the first one, patients have mild sensory and/or motor symptoms leading to difficulty in walking, but without need of assistance;
- In the second stage, there is sensorimotor polyneuropathy and assistance for walking is required;
- In the last stage, the patients are wheelchair-bound or bedridden. Death occurs on average 12 years after the onset of the disease.

This classical progression of the disease is deeply influenced by the presence of amyloidotic cardiac disease; therefore, survival in the variants with prevalent cardiological involvement is generally much shorter. For example, in ATTRI68L amyloidosis, the most frequent cardiac variant in Italy, survival is estimated to be 37% at 5 years from onset [16,17].

Liver transplantation (LT) and combined heart–liver transplantation (in case of severe cardiomyopathy, ATTR-CM) were introduced in the 1990s as the first treatment for ATTRv amyloidosis and remained for two decades as the only therapies available with clear clinical evidence. However, in many patients with non-V30M mutations, the benefits of LT have never been convincing. Although more slowly, the disease seems to progress even after LT, partly due to the toxic effect exerted by the already deposited amyloid and partly because the wild-type amyloid produced by the transplanted liver can continue to increase the accumulations of amyloid already present [18,19].

In the last decade, various disease-modifying therapies have been developed, and moreover, the awareness of being in front of a multisystem pathology that requires a multidisciplinary approach has also improved the treatment of symptoms [10,20]. In parallel, the increased knowledge and attention to the disease have also reduced the diagnostic delay, favoring an earlier start of therapies [4].

In this report, we provide an overview of available and emerging drugs for ATTRv amyloidosis, which have made the LT procedure now obsolete. Different targeted therapies, which aim to delay the deposition of misfolded TTR at various stages of the amyloidogenic process, have already obtained market access and some others are on the horizon [9,21,22].

The actual scenario of current pharmaceutical approaches for ATTRv amyloidosis includes five main groups:

- TTR stabilizers
- TTR mRNA silencers
- TTR fibril disruptors
- Inhibitor of TTR fibril seeding
- Gene therapy

## 2. TTR Stabilizers

### 2.1. Tafamidis

Tafamidis (Vyndaqel, Pfizer, New York, NY, USA) is a small molecule that is able to stabilize both variant TTR and wild-type TTR (ATTRwt), thus preventing tetramer dissociation [23]. It was the first specific drug approved for stage 1 ATTR-PN based on an 18-month, double-blind, placebo-controlled study, followed by an open label extension [24,25]. In the 18-month study, tafamidis treatment induced a significant reduction in worsening of most neurological variables in patients with early-stage V30M [24]. The extension study showed that long-term tafamidis was well-tolerated, with the reduced rate of neurological deterioration maintained over 30 months. Notably, initiation of therapy in the early stages of the disease induces a greater benefit [25].

Afterward, several clinical trials supported these findings, showing TTR stabilization in 89–100% of patients treated, confirming remarkable results in V30M patients and early disease stages. On the other hand, tafamidis does not seem able to stop disease progression in all the ATTR variants, especially in patients with advanced disease, rather slows worsening in V30M and non-V30M [26,27]. A longitudinal multicenter study in a non-endemic area reported that tafamidis is generally well-tolerated, even for long periods, but neuropathy and cardiomyopathy progress in a significant proportion of patients despite treatment [28].

Early treatment with tafamidis for up to 5.5 years further provided evidence of long-term benefits for patients with V30M, with no relevant safety issues [29]. In a population of ATTRv patients with a variety of pathogenetic V30M and non-V30M and a wide range of age and severity of neuropathy, tafamidis was well-tolerated, but did not prevent the steady progression in the long term in all the patients [30]. Similar results were reported in non-V30M patients treated with tafamidis up to 6 years. Neuropathy progression was observed across all efficacy measures evaluated [31]. A single center retrospective study of 210 tafamidis-treated patients showed that 34% of the patients had an arrest of disease progression and 36% had a partial response, whereas 30% appeared to have no benefit from the treatment. Disease severity, sex, and TTR concentration at the beginning of therapy were the most relevant predictors of response [32]. However, mortality data emerged in a recent study showed that approximately 85% of V30M patients and 75% of non-V30M patients were alive after 9 and 8 years of treatment with tafamidis, respectively (11 and 14 years after disease onset, respectively) [33]. This outcome is very encouraging, considering that life expectancy in untreated patients is about 10 years [34].

In conclusion, ATTR-PN in advanced stage at baseline is associated with poor response to tafamidis. The treatment is more effective in V30M than in non-V30M variants, especially regarding the progression of the polyneuropathy, but in terms of survival, both groups may benefit from tafamidis.

With regard to ATTR-CM, tafamidis is one of the most studied drugs and appears to be able to slow the progression of cardiac disease. In a randomized, double-blind, placebo-controlled, phase 3 study including patients with biopsy-proven ATTR-CM due to ATTRv or ATTRwt, 33-month tafamidis treatment was associated with reduction of all-cause mortality and cardiovascular-related hospitalizations. An analysis of survival curve showed a better trajectory of treated group, starting after 18 months of treatment [35].

To date, many regulatory agencies have approved the use of tafamidis in ATTR-PN in Europe, USA, Asia, and Latin America. In 2019, the U.S. Food and Drug Administration (FDA) approved tafamidis for ATTR-CM based on data from the abovementioned trial.

### 2.2. Diflunisal

In a cohort of patients with ATTRv amyloidosis, the use of diflunisal, a non-steroidal anti-inflammatory drug, compared with placebo for 2 years, reduced the progression of neuropathy and preserved quality of life irrespective of severity of disease at baseline and mutation [36]. Following studies have extended the benefits of diflunisal in maintaining cardiac and autonomic function and have confirmed its safety in long-term use [37,38]. However, diflunisal is not approved for ATTRv amyloidosis and can be used only “off-label”; consequently, it can only represent an alternative option in the event that approved therapies are ineffective or unavailable. A very interesting aspect of diflunisal is its very low cost compared to the currently approved drugs for ATTRv amyloidosis. Unfortunately, some typical adverse events of non-steroidal anti-inflammatory drugs, such as erosive gastritis and gastrointestinal bleeding, have been reported [39].

### 2.3. Epigallocatechin-3-Gallate (EGCG)

Among TTR stabilizers, EGCG, the major catechin found in green tea, showed promising results in different studies. Similarly to tafamidis, EGCG stabilizes ATTRwt and ATTRv, binding to a distinct site. EGCG appears to be able to prevent TTR aggregation and fibril formation in vitro [40], and to disrupt pre-formed amyloid fibrils in vitro and in animal models for ATTRv [41]. Two single-center observational studies reported a positive result of green tea consumption in patients affected by ATTRv and ATTRwt amyloidosis with cardiomyopathy [42,43]. In particular, in a cohort of 14 patients with ATTRwt and ATTRv cardiomyopathy, who presented various degrees of thickening of the ventricular walls, after 12 months no increase in left ventricular wall thickness and left ventricular myocardial mass was observed by echocardiography [42].

### 2.4. Tolcapone

Tolcapone is a selective, potent, and reversible nitrocatechol-type inhibitor of the enzyme catechol-*O*-methyltransferase approved for Parkinson disease [44]. Oral intake of tolcapone showed good efficacy in stabilizing the TTR tetramer in 82% of subjects taking 200 mg in a single dose and in 93% of those taking three 100 mg doses with 4-hour intervals. The drug was well-tolerated and clinical efficacy data are not yet available [45]. In addition, since tolcapone binds and stabilizes three unstable leptomeningeal TTR variants such as A25T, V30G and Y144C and because it crosses the blood–brain barrier, it may be the first specific drug for leptomeningeal amyloidosis [46].

### 2.5. AG10

AG10 is a potent, highly selective, kinetic stabilizer of TTR that shows interesting pharmaceutical properties, including good oral bioavailability [47]. A randomized, double-blind, placebo-controlled study in ATTR-CM patients with chronic heart failure showed that AG10 administration was well-tolerated and induced a significant stabilization of TTR [48]. Oral intake of AG10 appeared to be safe and well-tolerated in healthy adult volunteers and completely stabilize TTR across the dosing interval, and was effective for either ATTRv and ATTRwt [49]. The clinical efficacy of AG10 is not yet known; however, two clinical trials are ongoing, including a phase 3, randomized, double-blind, placebo-controlled study of the efficacy and safety of AG10 in subjects with symptomatic transthyretin amyloid cardiomyopathy (ATTRIBUTE-CM) that is still recruiting (ClinicalTrials.gov identifier: NCT03860935).

Moreover, a phase 3, randomized, double-blind, placebo-controlled study with AG10 is going to start to evaluate the efficacy and safety in subjects with ATTRv polyneuropathy (ATTRibute-PN; ClinicalTrials.gov identifier: NCT04418024).

### 2.6. Benzbromarone (BBM)

BBM is a uricosuric agent that acts as a non-competitive inhibitor of xanthine oxidase [50]. It represents a good alternative to allopurinol in the treatment of gout, thanks to its good tolerability [51]. BBM acts similarly to diflunisal, making TTR more resistant to urea denaturation. It binds TTR with a similar affinity to tolcapone, tafamidis, and diflunisal, forming a BBM-TTR intermonomer that stabilizes the TTR tetramer. The ability of this molecule to inhibit fibrillogenesis, compared to that of the other already-described stabilizers of TTR, suggests that it can be considered as a promising drug. However, the effectiveness of BBM has not been clinically tested; therefore, further studies are needed [52].

## 3. TTR mRNA Silencers

TTR gene silencing therapy with an antisense oligonucleotide (ASO) (inotersen) or a small interfering RNA (siRNA) (patisiran) is a recent, more promising therapeutic strategy for ATTRv amyloidosis. TTR silencers provide a real therapeutic revolution, showing evidence that disease progression can be slowed and perhaps reversed [53,54].

### 3.1. Inotersen

Inotersen is a second generation 2′-*O*-(2-methoxyethyl)-modified ASO, which is complimentary to a region in the 3′-untranslated region of the human wild-type and all known amyloidogenic variant TTR mRNA. It binds to mRNA mimicking the DNA/RNA complex, leading to RNase H1-mediated degradation of TTR mRNA, thus preventing hepatic TTR production [55]. Inotersen, administered subcutaneously every week, is able to inhibit the hepatic production of both ATTRv and ATTRwt. European Medical Agency (EMA) and FDA have recently approved it for the treatment of stage 1 and 2 polyneuropathies in patients with ATTRv (Tegsedi, Ackea Therapeutics, Boston, MA, USA). An international, randomized, double-blind, placebo-controlled, phase 3 trial (NEURO-TTR) assessed the safety and efficacy of this drug in ATTRv amyloidosis. The study reported a mean reduction of 74% in serum TTR. There have been some cases of renal dysfunction and thrombocytopenia; therefore, close monitoring of complete blood count and renal function is recommended [53]. A very recent, open, uncontrolled study reported that long-term treatment with inotersen is safe, effective, and potentially able to reverse amyloid burden in transthyretin amyloid cardiomyopathy [56]. TTR gene silencing therapy with inotersen could also be a treatment option in ATTRv patients who experienced disease progression after LT [57].

### 3.2. ION-682884

Very recently, another ASO seems to be even more promising than inotersen. ION-682884 is a second-generation drug targeted to TTR that has been covalently bound to tri-antennary *N*-acetyl galactosamine (GalNac), a high affinity ligand for a specific asialoglycoprotein receptor at the hepatic level. This GalNac approach results in increased ASO delivery to hepatocytes and raised ASO potency at least 10-fold in mice. ION-682884 was found to be more effective than unconjugated ASO inotersen in inhibiting TTR mRMN expression in TTR transgenic mice [58]. These results strongly support GalNac conjugation as a novel strategy to increase ASO potency, thus providing also the potential for improving safety and compliance, since subcutaneous administration is every four weeks. A phase 1/2 study to evaluate the safety and tolerability, as well as the pharmacokinetic (PK) and pharmacodynamic (PD) profiles, of ION-682884 in healthy volunteers and ATTRv patients has been initiated (ClinicalTrials.gov identifier: NCT03728634), and a phase 3 study is also ongoing and currently recruiting patients.

### 3.3. Patisiran

Patisiran is a small, double-stranded interfering RNA encapsulated in a lipid nanoparticle. It is delivered to the intracellular compartment of hepatocytes, where it selectively targets TTR mRNA, reducing both ATTRv and ATTRwt production [59]. The largest phase 3 trial for ATTRv polyneuropathy (APOLLO) showed the efficacy and safety profile of patisiran, infused intravenously every three weeks, over 18 months of treatment, with disease progression halted or regressed in some outcome measures. The study reported a mean reduction of 81% in serum TTR level [54]. As in the case of inotersen treatment, reduction in serum TTR correlated with depletion in retinol-binding protein and vitamin A; therefore, supplementation of vitamin A is required. While not a primary objective of the APOLLO study, an analysis of several cardiac parameters in a pre-specified cardiac subpopulation suggested a beneficial effect on cardiomyopathy [60].

Patisiran has gained both EMA and FDA approval for the treatment of adult patients with ATTR-PN (Onpattro, Alnylam, Cambridge, MA, USA).

More recently, a 24-month, phase 2, open-label extension study, conducted on 27 patients, confirmed the good tolerability and showed an improvement in the modified Neuropathy Impairment Score plus 7 (mNIS+7) of nearly 7 points in 24 months. Motor and autonomic function, as well as quality of life, remained stable, whereas an improvement in nerve-fiber density was documented by skin biopsies [61].

A comparative study between patisiran and inotersen, based on data from previously published studies, recently emphasized the greater efficacy of patisiran. In particular, better results were highlighted in mNIS+7 used in the IONIS trial [53] and Norfolk quality of life questionnaire-diabetic neuropathyas well as in the number of patients with improvement or no change from baseline on polyneuropathy disability score [62].

### 3.4. Vutrisiran

The siRNA pipeline of Alnylam also includes a phase 3 study to evaluate the efficacy and safety of vutrisiran, an RNA interference drug administered subcutaneously once every 3 months (CinicalTrials.gov identifier: NCT03759379). In a phase 1 randomized, single-blind, placebo-controlled study, vutrisiran treatment achieved potent and sustained TTR reduction in a dose-dependent manner for ≥90 days post-dose. Vutrisiran also had a good safety profile [63].

## 4. Fibril Disruptors

### 4.1. Doxycycline

Doxycycline, a tetracycline antibiotic, was studied in an ATTRv mouse model where it disrupted amyloid deposits [64]. Although there are no clinical trials confirming its efficacy in ATTRv amyloidosis, the safety of doxycycline along with bortezomib-based chemotherapy was confirmed in a recent phase 2 trial in patients with amyloid light-chain amyloidosis [65].

### 4.2. Tauroursodeoxycholic Acid (TUDCA)

TUDCA, a biliary acid, significantly reduced fibril aggregation in transgenic mice [66].

Combined doxycycline and TUDCA administration to mice with amyloid deposition resulted in a synergistic effect in lowering TTR deposition and associated biomarkers [67]. A combination of oral doxycycline and TUDCA stabilized the disease for at least 1 year in ATTRv and ATTRwt amyloidosis [68]. The use of the two drugs in combination has also been reported in ATTR cardiac amyloidosis. The treatment was overall well-tolerated, although six out of fifty-three patients had to discontinue therapy due to gastrointestinal and dermatological side effects. Furthermore, treated patients showed no significant change in New York Heart Association functional class, cardiac biomarkers, or echocardiographic parameters during a median follow-up of 22 months [69].

A randomized, phase 3 study of doxycycline/TUDCA plus standard supportive therapy versus standard supportive therapy alone in cardiac amyloidosis is ongoing (ClinicalTrials.gov identifier: NCT03481972).

### 4.3. Monoclonal Antibodies

Monoclonal antibodies represent one of the latest therapeutic options for ATTRv amyloidosis. During TTR fibrillation, new epitopes (cryptic epitopes) are exposed on the molecular surface with a conformational change of TTR [70]. Anti-TTR antibodies may attach to amyloid fibrils and pre-fibrillar TTR [71]. Targeting ATTR amyloid fibrils via antibody induces complement activation and phagocytosis of fibrils present in the deposits. Several monoclonal antibodies against TTR epitopes have been tested in vitro as potential new drugs [72,73,74].

Dezamizumab is a fully humanized monoclonal IgG1 anti- serum amyloid P component antibody that triggers immunotherapeutic clearance of amyloid. Amyloid load reduction was reported in the liver, spleen, and kidney following its administration in AL and ATTR systemic amyloidosis [75].

Finally, a phase 1 clinical trial with the intravenous infusion of PRX004 (an investigational monoclonal antibody targeting a novel epitope on ATTR fibrils) is ongoing (ClinicalTrials.gov identifier: NCT03336580).

## 5. Inhibitor of TTR Fibril Seeding

One of the mechanisms that appears to be the cause of failure of liver transplantation as well as TTR stabilizers in advanced stages of the disease is the ability of amyloid seeding to promote fibrillogenesis. If mutant amyloid seeds remain in the tissues, they are able to convert TTR, perpetuating the disease. This mechanism could be particularly relevant in patients with cardiac amyloid deposits. Therefore, an additional strategy to halt protein aggregation may be the use of peptides designed to complement structures of TTR fibrils, thereby inhibiting TTR fibril seeding. Among these new potential drugs, TabFH2 blockers are the most promising [76,77]. In two *Drosophila* models of neuropathic ATTR, the peptide inhibitor TabFH2 was found to be more effective compared with diflunisal, resulting in motor improvement and reduction of TTR deposition [78].

## 6. Gene Therapy

The “new frontier” in therapeutics is now Clustered Regularly Interspaced Short Palindromic Repeats (CRISPR)/Cas9 genome editing system to cut and repair a specific target sequence of DNA in a genome. An investigational new drug called NTLA-2001 is the first CRISPR/Cas9 therapy for the treatment of ATTRv amyloidosis. Recently, Intellia Therapeutics completed a 12-month durability study of its lead lipid nanoparticle formulation, obtaining an average reduction of >95% of serum TTR in non-human primates. A phase 1, open-label study to evaluate the safety, tolerability, PK, and PD of this novel agent is nearly underway.

## 7. Concluding Remarks

The progressive nature of a devastating disease such as ATTRv amyloidosis emphasizes the need for timely diagnosis and intervention. Several approved treatments and emerging options capable of reducing ATTRv and ATTRwt production as well as neutralizing fibril formation are now available. The type of TTR mutation and disease stage, as well as the age and the general clinical condition of the patient should be carefully considered in order to select the best treatment option.

The accessibility of many effective treatments also raises issues regarding genetic screening and the management of asymptomatic carriers. Protein TTR misfolding and tissue deposition of amyloid start before the appearance of symptoms in ATTRv amyloidosis. Future studies are needed to determine if an earlier initiation of treatment would be able to improve the clinical course of the disease. For this aim, new biomarkers can help in monitoring the shift from asymptomatic stage to stage 1 [79].

## References

[1] Adams D., Ando Y., Beirão J.M., Coelho T., Gertz M.A., Gillmore J.D., Hawkins P.N., Lousada I., Suhr O.B., Merlini G. (2020). Expert consensus recommendations to improve diagnosis of ATTR amyloidosis with polyneuropathy. J. Neurol..

[2] Schmidt H.H., Waddington-Cruz M., Botteman M.F., Carter J.A., Chopra A.S., Hopps M., Stewart M., Fallet S., Amass L. (2018). Estimating the global prevalence of transthyretin familial amyloid polyneuropathy: ATTR-FAP Global Prevalence. Muscle Nerve.

[3] Waddington-Cruz M., Schmidt H., Botteman M.F., Carter J.A., Stewart M., Hopps M., Fallet S., Amass L. (2019). Epidemiological and clinical characteristics of symptomatic hereditary transthyretin amyloid polyneuropathy: A global case series. Orphanet J. Rare Dis..

[4] Russo M., Obici L., Bartolomei I., Cappelli F., Luigetti M., Fenu S., Cavallaro T., Chiappini M.G., Gemelli C., Pradotto L.G. (2020). ATTRv amyloidosis Italian Registry: Clinical and epidemiological data. Amyloid.

[5] Koike H., Sobue G. (2012). Late-onset familial amyloid polyneuropathy in Japan. Amyloid.

[6] Mariani L., Lozeron P., Théaudin M., Mincheva Z., Signate A., Ducot B., Algalarrondo V., Denier C., Adam C., Nicolas G. (2015). Genotype–phenotype correlation and course of transthyretin familial amyloid polyneuropathies in France. Ann. Neurol..

[7] Russo M., Mazzeo A., Stancanelli C., Di Leo R., Gentile L., Di Bella G., Minutoli F., Baldari S., Vita G. (2012). Transthyretin-related familial amyloidotic polyneuropathy: Description of a cohort of patients with Leu64 mutation and late onset. J. Peripher. Nerv. Syst..

[8] Parman Y., Adams D., Obici L., Galán L., Guergueltcheva V., Suhr O.B., Coelho T. (2016). Sixty years of transthyretin familial amyloid polyneuropathy (TTR-FAP) in Europe: Where are we now? A European network approach to defining the epidemiology and management patterns for TTR-FAP. Curr. Opin. Neurol..

[9] Vita G., Vita G.L., Stancanelli C., Gentile L., Russo M., Mazzeo A. (2019). Genetic neuromuscular disorders: Living the era of a therapeutic revolution. Part 1: Peripheral neuropathies. Neurol. Sci..

[10] Adams D., Koike H., Slama M., Coelho T. (2019). Hereditary transthyretin amyloidosis: A model of medical progress for a fatal disease. Nat. Rev. Neurol..

[11] Stancanelli C., Gentile L., Di Bella G., Minutoli F., Russo M., Vita G., Mazzeo A. (2017). Phenotypic variability of TTR Val122Ile mutation: A Caucasian patient with axonal neuropathy and normal heart. Neurol. Sci..

[12] Gentile L., Di Bella G., Minutoli F., Cucinotta F., Obici L., Mussinelli R., Arimatea I., Russo M., Toscano A., Vita G. (2020). Description of a large cohort of Caucasian patients with V122I ATTRV amyloidosis: Neurological and cardiological features. J. Peripher. Nerv. Syst..

[13] Plante-Bordeneuve V. (2018). Transthyretin familial amyloid polyneuropathy: An update. J. Neurol..

[14] Luigetti M., Romozzi M., Bisogni G., Cardellini D., Cavallaro T., Di Paolantonio A., Fabrizi G.M., Fenu S., Gentile L., Grandis M. (2020). hATTR Pathology: Nerve Biopsy Results from Italian Referral Centers. Brain Sci..

[15] Coutinho P., Martins da Silva A., Lopes Lima J., Glenner G., Costa P., de Freitas A. (1980). Forty years of experience with type I amyloid neuropathy. Review of 483 cases. Amyloid and Amyloidosis.

[16] Maurer M.S., Bokhari S., Damy T., Dorbala S., Drachman B.M., Fontana M., Grogan M., Kristen A.V., Lousada I., Nativi-Nicolau J. (2019). Expert Consensus Recommendations for the Suspicion and Diagnosis of Transthyretin Cardiac Amyloidosis. Circ. Heart Fail..

[17] Gagliardi C., Perfetto F., Lorenzini M., Ferlini A., Salvi F., Milandri A., Quarta C.C., Taborchi G., Bartolini S., Frusconi S. (2018). Phenotypic profile of Ile68Leu transthyretin amyloidosis: An underdiagnosed cause of heart failure: Ile68Leu transthyretin amyloidosis. Eur. J. Heart Fail..

[18] Benson M.D. (2013). Liver transplantation and transthyretin amyloidosis: Liver Transplantation for ATTR. Muscle Nerve.

[19] Yamashita T., Ando Y., Okamoto S., Misumi Y., Hirahara T., Ueda M., Obayashi K., Nakamura M., Jono H., Shono M. (2012). Long-term survival after liver transplantation in patients with familial amyloid polyneuropathy. Neurology.

[20] Russo M., Vita G.L., Stancanelli C., Mazzeo A., Vita G., Messina S. (2016). Parenteral nutrition improves nutritional status, autonomic symptoms and quality of life in transthyretin amyloid polyneuropathy. Neuromuscul. Disord..

[21] Müller M.L., Butler J., Heidecker B. (2020). Emerging therapies in transthyretin amyloidosis—A new wave of hope after years of stagnancy?. Eur. J. Heart Fail..

[22] Hough A., Wearden J., de Almeida K., Kaiser S. (2020). Review of Transthyretin Silencers, Stabilizers, and Fibril Removal Agents in the Treatment of Transthyretin Cardiac Amyloid. Curr. Cardiol. Rep..

[23] Bulawa C.E., Connelly S., DeVit M., Wang L., Weigel C., Fleming J.A., Packman J., Powers E.T., Wiseman R.L., Foss T.R. (2012). Tafamidis, a potent and selective transthyretin kinetic stabilizer that inhibits the amyloid cascade. Proc. Natl. Acad. Sci. USA.

[24] Coelho T., Maia L.F., Martins da Silva A., Waddington Cruz M., Plante-Bordeneuve V., Lozeron P., Suhr O.B., Campistol J.M., Conceicao I.M., Schmidt H.H.-J. (2012). Tafamidis for transthyretin familial amyloid polyneuropathy: A randomized, controlled trial. Neurology.

[25] Coelho T., Maia L.F., da Silva A.M., Cruz M.W., Planté-Bordeneuve V., Suhr O.B., Conceiçao I., Schmidt H.H.-J., Trigo P., Kelly J.W. (2013). Long-term effects of tafamidis for the treatment of transthyretin familial amyloid polyneuropathy. J. Neurol..

[26] Lozeron P., Théaudin M., Mincheva Z., Ducot B., Lacroix C., Adams D., French Network for FAP (CORNAMYL) (2013). Effect on disability and safety of Tafamidis in late onset of Met30 transthyretin familial amyloid polyneuropathy. Eur. J. Neurol..

[27] Merlini G., Planté-Bordeneuve V., Judge D.P., Schmidt H., Obici L., Perlini S., Packman J., Tripp T., Grogan D.R. (2013). Effects of Tafamidis on Transthyretin Stabilization and Clinical Outcomes in Patients with Non-Val30Met Transthyretin Amyloidosis. J. Cardiovasc. Trans. Res..

[28] Cortese A., Vita G., Luigetti M., Russo M., Bisogni G., Sabatelli M., Manganelli F., Santoro L., Cavallaro T., Fabrizi G.M. (2016). Monitoring effectiveness and safety of Tafamidis in transthyretin amyloidosis in Italy: A longitudinal multicenter study in a non-endemic area. J. Neurol..

[29] Waddington Cruz M., Amass L., Keohane D., Schwartz J., Li H., Gundapaneni B. (2016). Early intervention with tafamidis provides long-term (5.5-year) delay of neurologic progression in transthyretin hereditary amyloid polyneuropathy. Amyloid.

[30] Planté-Bordeneuve V., Gorram F., Salhi H., Nordine T., Ayache S.S., Le Corvoisier P., Azoulay D., Feray C., Damy T., Lefaucheur J.-P. (2017). Long-term treatment of transthyretin familial amyloid polyneuropathy with tafamidis: A clinical and neurophysiological study. J. Neurol..

[31] Barroso F.A., Judge D.P., Ebede B., Li H., Stewart M., Amass L., Sultan M.B. (2017). Long-term safety and efficacy of tafamidis for the treatment of hereditary transthyretin amyloid polyneuropathy: Results up to 6 years. Amyloid.

[32] Monteiro C., Mesgazardeh J.S., Anselmo J., Fernandes J., Novais M., Rodrigues C., Brighty G.J., Powers D.L., Powers E.T., Coelho T. (2019). Predictive model of response to tafamidis in hereditary ATTR polyneuropathy. JCI Insight.

[33] Merlini G., Coelho T., Waddington Cruz M., Li H., Stewart M., Ebede B. (2020). Evaluation of Mortality During Long-Term Treatment with Tafamidis for Transthyretin Amyloidosis with Polyneuropathy: Clinical Trial Results up to 8.5 Years. Neurol. Ther..

[34] Ando Y., Coelho T., Berk J.L., Cruz M.W., Ericzon B.-G., Ikeda S., Lewis W.D., Obici L., Planté-Bordeneuve V., Rapezzi C. (2013). Guideline of transthyretin-related hereditary amyloidosis for clinicians. Orphanet J. Rare Dis..

[35] Maurer M.S., Schwartz J.H., Gundapaneni B., Elliott P.M., Merlini G., Waddington-Cruz M., Kristen A.V., Grogan M., Witteles R., Damy T. (2018). Tafamidis Treatment for Patients with Transthyretin Amyloid Cardiomyopathy. N. Engl. J. Med..

[36] Berk J.L., Suhr O.B., Obici L., Sekijima Y., Zeldenrust S.R., Yamashita T., Heneghan M.A., Gorevic P.D., Litchy W.J., Wiesman J.F. (2013). Repurposing Diflunisal for Familial Amyloid Polyneuropathy: A Randomized Clinical Trial. JAMA.

[37] Sekijima Y., Tojo K., Morita H., Koyama J., Ikeda S. (2015). Safety and efficacy of long-term diflunisal administration in hereditary transthyretin (ATTR) amyloidosis. Amyloid.

[38] Takahashi R., Ono K., Shibata S., Nakamura K., Komatsu J., Ikeda Y., Ikeda T., Samuraki M., Sakai K., Iwasa K. (2014). Efficacy of diflunisal on autonomic dysfunction of late-onset familial amyloid polyneuropathy (TTR Val30Met) in a Japanese endemic area. J. Neurol. Sci..

[39] Ikram A., Donnelly J.P., Sperry B.W., Samaras C., Valent J., Hanna M. (2018). Diflunisal tolerability in transthyretin cardiac amyloidosis: A single center’s experience. Amyloid.

[40] Ferreira N., Cardoso I., Domingues M.R., Vitorino R., Bastos M., Bai G., Saraiva M.J., Almeida M.R. (2009). Binding of epigallocatechin-3-gallate to transthyretin modulates its amyloidogenicity. FEBS Lett..

[41] Ferreira N., Saraiva M.J., Almeida M.R. (2012). Epigallocatechin-3-Gallate as a Potential Therapeutic Drug for TTR-Related Amyloidosis: “In Vivo” Evidence from FAP Mice Models. PLoS ONE.

[42] Kristen A.V., Lehrke S., Buss S., Mereles D., Steen H., Ehlermann P., Hardt S., Giannitsis E., Schreiner R., Haberkorn U. (2012). Green tea halts progression of cardiac transthyretin amyloidosis: An observational report. Clin. Res. Cardiol..

[43] aus dem Siepen F., Buss S.J., Andre F., Seitz S., Giannitsis E., Steen H., Katus H.A., Kristen A.V. (2015). Extracellular remodeling in patients with wild-type amyloidosis consuming epigallocatechin-3-gallate: Preliminary results of T1 mapping by cardiac magnetic resonance imaging in a small single center study. Clin. Res. Cardiol..

[44] Antonini A. (2008). COMT inhibition with tolcapone in the treatment algorithm of patients with Parkinson’s disease (PD): Relevance for motor and non-motor features. Neuropsychiatr. Dis. Treat..

[45] Gamez J., Salvadó M., Reig N., Suñé P., Casasnovas C., Rojas-Garcia R., Insa R. (2019). Transthyretin stabilization activity of the catechol-O-methyltransferase inhibitor tolcapone (SOM0226) in hereditary ATTR amyloidosis patients and asymptomatic carriers: Proof-of-concept study. Amyloid.

[46] Pinheiro F., Varejão N., Esperante S., Santos J., Velázquez-Campoy A., Reverter D., Pallarès I., Ventura S. (2020). Tolcapone, a potent aggregation inhibitor for the treatment of familial leptomeningeal amyloidosis. FEBS J..

[47] Penchala S.C., Connelly S., Wang Y., Park M.S., Zhao L., Baranczak A., Rappley I., Vogel H., Liedtke M., Witteles R.M. (2013). AG10 inhibits amyloidogenesis and cellular toxicity of the familial amyloid cardiomyopathy-associated V122I transthyretin. Proc. Natl. Acad. Sci. USA.

[48] Judge D.P., Heitner S.B., Falk R.H., Maurer M.S., Shah S.J., Witteles R.M., Grogan M., Selby V.N., Jacoby D., Hanna M. (2019). Transthyretin Stabilization by AG10 in Symptomatic Transthyretin Amyloid Cardiomyopathy. J. Am. Coll. Cardiol..

[49] Fox J.C., Hellawell J.L., Rao S., O’Reilly T., Lumpkin R., Jernelius J., Gretler D., Sinha U. (2020). First-in-Human Study of AG10, a Novel, Oral, Specific, Selective, and Potent Transthyretin Stabilizer for the Treatment of Transthyretin Amyloidosis: A Phase 1 Safety, Tolerability, Pharmacokinetic, and Pharmacodynamic Study in Healthy Adult Volunteers. Clin. Pharmacol. Drug Dev..

[50] Sinclair D.S., Fox I.H. (1975). The pharmacology of hypouricemic effect of benzbromarone. J. Rheumatol..

[51] Reinders M.K., van Roon E.N., Jansen T.L.T.A., Delsing J., Griep E.N., Hoekstra M., van de Laar M.a.F.J., Brouwers J.R.B.J. (2009). Efficacy and tolerability of urate-lowering drugs in gout: A randomised controlled trial of benzbromarone versus probenecid after failure of allopurinol. Ann. Rheum. Dis..

[52] Cotrina E.Y., Oliveira Â., Leite J.P., Llop J., Gales L., Quintana J., Cardoso I., Arsequell G. (2020). Repurposing Benzbromarone for Familial Amyloid Polyneuropathy: A New Transthyretin Tetramer Stabilizer. Int. J. Mol. Sci..

[53] Benson M.D., Waddington-Cruz M., Berk J.L., Polydefkis M., Dyck P.J., Wang A.K., Planté-Bordeneuve V., Barroso F.A., Merlini G., Obici L. (2018). Inotersen Treatment for Patients with Hereditary Transthyretin Amyloidosis. N. Engl. J. Med..

[54] Adams D., Gonzalez-Duarte A., O’Riordan W.D., Yang C.-C., Ueda M., Kristen A.V., Tournev I., Schmidt H.H., Coelho T., Berk J.L. (2018). Patisiran, an RNAi Therapeutic, for Hereditary Transthyretin Amyloidosis. N. Engl. J. Med..

[55] Ackermann E.J., Guo S., Benson M.D., Booten S., Freier S., Hughes S.G., Kim T.-W., Jesse Kwoh T., Matson J., Norris D. (2016). Suppressing transthyretin production in mice, monkeys and humans using 2nd-Generation antisense oligonucleotides. Amyloid.

[56] Dasgupta N.R., Rissing S.M., Smith J., Jung J., Benson M.D. (2020). Inotersen therapy of transthyretin amyloid cardiomyopathy. Amyloid.

[57] Moshe-Lilie O., Dimitrova D., Heitner S.B., Brannagan T.H., Zivkovic S., Hanna M., Masri A., Polydefkis M., Berk J.L., Gertz M.A. (2020). TTR gene silencing therapy in post liver transplant hereditary ATTR amyloidosis patients. Amyloid.

[58] Prakash T.P., Graham M.J., Yu J., Carty R., Low A., Chappell A., Schmidt K., Zhao C., Aghajan M., Murray H.F. (2014). Targeted delivery of antisense oligonucleotides to hepatocytes using triantennary N-acetyl galactosamine improves potency 10-fold in mice. Nucleic Acids Res..

[59] Suhr O.B., Coelho T., Buades J., Pouget J., Conceicao I., Berk J., Schmidt H., Waddington-Cruz M., Campistol J.M., Bettencourt B.R. (2015). Efficacy and safety of patisiran for familial amyloidotic polyneuropathy: A phase II multi-dose study. Orphanet J. Rare Dis..

[60] Solomon S.D., Adams D., Kristen A., Grogan M., González-Duarte A., Maurer M.S., Merlini G., Damy T., Slama M.S., Brannagan T.H. (2019). Effects of Patisiran, an RNA Interference Therapeutic, on Cardiac Parameters in Patients With Hereditary Transthyretin-Mediated Amyloidosis: Analysis of the APOLLO Study. Circulation.

[61] Coelho T., Adams D., Conceição I., Waddington-Cruz M., Schmidt H.H., Buades J., Campistol J., Berk J.L., Polydefkis M., Wang J.J. (2020). A phase II, open-label, extension study of long-term patisiran treatment in patients with hereditary transthyretin-mediated (hATTR) amyloidosis. Orphanet J. Rare Dis..

[62] Gorevic P., Franklin J., Chen J., Sajeev G., Wang J.C.H., Lin H. (2020). Indirect treatment comparison of the efficacy of patisiran and inotersen for hereditary transthyretin-mediated amyloidosis with polyneuropathy. Expert Opin. Pharm..

[63] Habtemariam B.A., Karsten V., Attarwala H., Goel V., Melch M., Clausen V.A., Garg P., Vaishnaw A.K., Sweetser M.T., Robbie G.J. (2020). Single-Dose Pharmacokinetics and Pharmacodynamics of Transthyretin Targeting N-acetylgalactosamine–Small Interfering Ribonucleic Acid Conjugate, Vutrisiran, in Healthy Subjects. Clin. Pharmacol. Ther..

[64] Cardoso I., Saraiva M.J. (2006). Doxycycline disrupts transthyretin amyloid: Evidence from studies in a FAP transgenic mice model. FASEB J..

[65] D’Souza A., Szabo A., Flynn K.E., Dhakal B., Chhabra S., Pasquini M.C., Weihrauch D., Hari P.N. (2020). Adjuvant Doxycycline to Enhance Anti-Amyloid Effects: Results from the DUAL Phase 2 Trial. SSRN J..

[66] Macedo B., Batista A.R., Ferreira N., Almeida M.R., Saraiva M.J. (2008). Anti-apoptotic treatment reduces transthyretin deposition in a transgenic mouse model of Familial Amyloidotic Polyneuropathy. Biochim. Biophys. Acta.

[67] Cardoso I., Martins D., Ribeiro T., Merlini G., Saraiva M. (2010). Synergy of combined Doxycycline/TUDCA treatment in lowering Transthyretin deposition and associated biomarkers: Studies in FAP mouse models. J. Transl. Med..

[68] Obici L., Cortese A., Lozza A., Lucchetti J., Gobbi M., Palladini G., Perlini S., Saraiva M.J., Merlini G. (2012). Doxycycline plus tauroursodeoxycholic acid for transthyretin amyloidosis: A phase II study. Amyloid.

[69] Karlstedt E., Jimenez-Zepeda V., Howlett J.G., White J.A., Fine N.M. (2019). Clinical Experience with the Use of Doxycycline and Ursodeoxycholic Acid for the Treatment of Transthyretin Cardiac Amyloidosis. J. Card. Fail..

[70] Goldsteins G., Persson H., Andersson K., Olofsson A., Dacklin I., Edvinsson A., Saraiva M.J., Lundgren E. (1999). Exposure of cryptic epitopes on transthyretin only in amyloid and in amyloidogenic mutants. Proc. Natl. Acad. Sci. USA.

[71] Su Y., Jono H., Torikai M., Hosoi A., Soejima K., Guo J., Tasaki M., Misumi Y., Ueda M., Shinriki S. (2012). Antibody therapy for familial amyloidotic polyneuropathy. Amyloid.

[72] Ando Y., Ueda M. (2017). Antibody therapy for transthyretin-related hereditary amyloid polyneuropathy: Another therapeutic option. Amyloid.

[73] Higaki J.N., Chakrabartty A., Galant N.J., Hadley K.C., Hammerson B., Nijjar T., Torres R., Tapia J.R., Salmans J., Barbour R. (2016). Novel conformation-specific monoclonal antibodies against amyloidogenic forms of transthyretin. Amyloid.

[74] Hosoi A., Su Y., Torikai M., Jono H., Ishikawa D., Soejima K., Higuchi H., Guo J., Ueda M., Suenaga G. (2016). Novel Antibody for the Treatment of Transthyretin Amyloidosis. J. Biol. Chem..

[75] Richards D.B., Cookson L.M., Barton S.V., Liefaard L., Lane T., Hutt D.F., Ritter J.M., Fontana M., Moon J.C., Gillmore J.D. (2018). Repeat doses of antibody to serum amyloid P component clear amyloid deposits in patients with systemic amyloidosis. Sci. Transl. Med..

[76] Saelices L., Chung K., Lee J.H., Cohn W., Whitelegge J.P., Benson M.D., Eisenberg D.S. (2018). Amyloid seeding of transthyretin by ex vivo cardiac fibrils and its inhibition. Proc. Natl. Acad. Sci. USA.

[77] Saelices L., Nguyen B.A., Chung K., Wang Y., Ortega A., Lee J.H., Coelho T., Bijzet J., Benson M.D., Eisenberg D.S. (2018). A pair of peptides inhibits seeding of the hormone transporter transthyretin into amyloid fibrils. J. Biol. Chem..

[78] Saelices L., Pokrzywa M., Pawelek K., Eisenberg D.S. (2018). Assessment of the effects of transthyretin peptide inhibitors in Drosophila models of neuropathic ATTR. Neurobiol. Dis..

[79] Vita G.L., Aguennouz M., Polito F., Oteri R., Russo M., Gentile L., Barbagallo C., Ragusa M., Rodolico C., Di Giorgio R.M. (2020). Circulating microRNAs Profile in Patients with Transthyretin Variant Amyloidosis. Front. Mol. Neurosci..

